# Development of a scale to measure intrapersonal factors influencing speaking up in the operating room

**DOI:** 10.1007/s40037-019-00529-4

**Published:** 2019-07-25

**Authors:** Serkan Toy, Rodrigo J. Daly Guris, Shirley S Duarte, Priyanka Dwivedi

**Affiliations:** 1 Department of Anesthesiology and Critical Care Medicine, Johns Hopkins Medicine, Baltimore, MD USA; 2 Department of Anesthesiology and Critical Care Medicine, Children’s Hospital of Philadelphia, Philadelphia, PA USA

**Keywords:** Speaking up in the operating room, Anaesthesiology residency education, Scale development, Self-efficacy, Outcome expectations, Assertive attitude

## Abstract

**Introduction:**

This paper reports on the development of a scale to measure intrapersonal factors (IPF) that may influence speaking up behaviour in the operating room.

**Methods:**

Participants were postgraduate year 2, 3, and 4 anaesthesiology residents and practising faculty anaesthesiologists at a large quaternary care academic hospital. Based on a literature review, the authors constructed the initial scale. Exploratory factor analysis was conducted to identify the underlying factor structure for the scale. A set of one-way ANOVAs and multiple ordinal regressions were carried out to provide additional validity evidence for the new scale.

**Results:**

Exploratory factor analysis indicated a three-factor solution accounting for 73% of the variance. The self-efficacy subscale included four items (Cronbach’s α = 0.86), and the social outcome expectations (Cronbach’s α = 0.86) and assertive attitude (Cronbach’s α = 0.67) subscales contained three items each. The effect of training level was significantly associated with self-efficacy (*p* < 0.001) and assertive attitude subscale scores (*p* < 0.001). Multiple ordinal regressions indicated that IPF predicted participants’ likelihood of speaking up in various hypothetical scenarios.

**Discussion:**

Our analyses provided initial evidence for the validity and reliability of a 10-item IPF scale. This instrument needs to be validated in other cohorts.

## What this paper adds

We addressed the questions whether a psychometrically sound scale could be developed to measure intrapersonal factors that influence decisions to speak up about patient management concerns in the operating room. Using exploratory factor analysis, a 10-item scale emerged with a three-factor solution (self-efficacy, social outcome expectations, and assertive attitude), which was sensitive to the participants’ level of training and predicted their likelihood of speaking up in various hypothetical scenarios. This study provides initial evidence for the validity and reliability of a 10-item scale, which attempts to measure three distinct aspects of the intrapersonal factors for speaking up in the OR.

## Introduction

It should be obvious that timely and well-coordinated, rapid team efforts are crucial for safe and effective patient care in the operating room (OR), especially when faced with unexpected events. That said, patient safety can be seriously threatened by breakdowns in communication between healthcare practitioners [1–5]. High-acuity, time-critical ORs, staffed by professionals from diverse disciplines with differing training and experience, are especially vulnerable to such errors.

Anaesthesiologists are responsible for the continual monitoring of patients during surgery, and often have the most detailed information about patients’ dynamic physiological conditions. Like each member of the OR team, anaesthesiologists are also in a position to notice patient safety concerns, and to help the OR team establish a shared understanding regarding continued care. Junior anaesthesiology residents, however, may hesitate to speak up due to various reasons [6–9]. Previous studies have identified aspects of organizational culture as well as a number of individual factors as the main barriers to speaking up [6–14].

A hierarchical team structure, institutional policies and support, and a perceived safety of speaking up are common organizational factors reported to influence speaking up behaviour [6–8, 12, 13]. There can also be the unintended effects of the hidden curriculum undermining the effectiveness of any explicit educational intervention [15, 16]. These are important to identify, as trainees often develop behaviours based on their interactions within a given cultural context, and more hierarchical ORs may not be conducive to developing effective speaking up behaviour for trainees.

Beyond environmental factors, however, individuals usually decide whether to speak up based on an internal thought process. Without a way of measuring what elements contribute to this intrapersonal process, it is difficult to develop well-informed and effective training efforts. Existing research has drawn on either personality traits or attribute constructs to tap into bravery and assertiveness [17] or agency and communion [14], or sought to adapt items from safety attitudes questionnaires [18] to measure confidence assertion [19]. To the best of our knowledge there is no scale specifically measuring intrapersonal factors (IPF) surrounding speaking up behaviour in the OR environment. Such an instrument could help identify opportunities for improvement and guide curriculum development efforts. The objective of this study is to develop a scale to assess intrapersonal variables contributing to the decision to speak up about patient management concerns in the OR.

In our review of the literature, we found no single unifying theoretical framework that seemed to adequately explain underlying individual factors that influence how one decides to speak up about a patient management concern under critical, time-pressured perioperative situations where a hierarchical culture may exist. Existing literature does indicate that a number of individual factors could be important in understanding why some people choose to speak up whereas others remain silent, even within the same cultural context. These factors, which are further characterized below, are termed self-efficacy, outcome expectancy, and assertiveness [8, 10–12, 14].

### Self-efficacy

Self-efficacy refers to the belief in one’s own ability to execute the courses of action necessary to successfully perform a given task [20]. Bandura (1977) suggests that individuals either attempt or avoid certain tasks depending on whether or not they believe that they possess the ability to attain the desired outcome. Once individuals decide to attempt a task, self-efficacy beliefs seem to affect how much effort they will invest and how long they will persist in accomplishing this task in the face of challenges [20].

Self-efficacy is considered to be task and context specific and is better measured within a particular context of functioning [21]. For example, a trainee might feel that he or she can effectively hand off a patient to a colleague, but may lack self-efficacy in his or her ability to communicate a patient management concern to a surgeon. Self-efficacy beliefs are not intentional statements about what someone will do, rather what they can do in a given task situation [22]. In summarizing findings from nine meta-analyses, Bandura and Locke (2003) concluded that evidence from diverse domains (academic success, psychological functioning, patient health outcomes, and sport performance) suggests that self-efficacy beliefs enhance motivation and performance [23].

### Outcome expectancy

Outcome expectancy, which is different than self-efficacy, refers to a person’s appraisal that a given behaviour will produce a certain outcome [20]. One may believe that a certain action is likely to produce a desired outcome while simultaneously lacking the self-efficacy to carry out this action. On the other hand, if one lacks the outcome expectancy belief that a behaviour will produce a beneficial outcome (whether psychological, physical, or social), one may not be motivated to carry out a task at all, even when possessing strong self-efficacy beliefs. In the context of the OR, specifically social outcome expectations might play an important role in deciding whether to speak up or remain silent about a patient management concern.

While some studies show that self-efficacy beliefs were better predictors of performance than outcome expectations [24], others found that outcome expectancy strengthened the intentions to perform a given behaviour [25], such as in predicting treatment response of cognitive behavioural therapy for public speaking fears within social anxiety disorder [26].

### Assertive attitude

Assertiveness has been described as effective communication of ideas without hesitation in a clear and direct fashion, even under seemingly stressful situations [27]. Several studies indicate that assertiveness is an important factor in speaking up behaviour in the OR environment, especially when hierarchical culture may cause trainees to hesitate voicing concerns [10–12]. Another study identifies agency (assertiveness and persistence) as a positive predictor of speaking up in acute care teams [14]. There is also evidence to suggest that the two-challenge rule and advocacy-inquiry communication methods provide useful frameworks for developing assertiveness in speaking up for anaesthesiology trainees [7, 9].

## Methods

### Participants and procedures

The study was performed in accordance with the ethical standards of the institutional and/or national research committee and with the 1964 Helsinki Declaration and its later amendments or comparable ethical standards. Our Institutional Review Board deemed this study exempt from informed consent and waived the requirement for documentation of consent (IRB file number: 00135217). Verbal or online informed consent was obtained from all individual participants included in the study. Participants were postgraduate year (PGY) 2, 3, and 4 anaesthesiology trainees and attending faculty anaesthesiologists at a large quaternary care academic hospital located in the East Coast of the United States. The questionnaire was available in print as well as in online format. We requested trainees to complete the printed version at the end of an unrelated didactic session. Those residents who were not available during the didactic session received an email with a link to the online version of the questionnaire. Attending faculty anaesthesiologists also received an email with a link to the online version of the questionnaire requesting their participation. All data collection was completed between April and June 2017.

### Questionnaire

Based on review of existing literature, a pool of initial items related to self-efficacy, outcome expectations (especially concerning social outcome expectations), and assertiveness was drafted to explore clinicians’ perspectives on speaking up in the OR. Three physicians and two educational researchers reviewed this draft to ensure face and content validity. The final scale used in the study included 20 items (7 self-efficacy, 6 outcome expectancy, 7 assertive attitude), including ‘I feel confident that I can recognize when something is amiss during a surgery’; ‘My team will view me as competent if I effectively express patient safety concerns in the OR’; and ‘I think it is better to defer to senior staff rather than voicing concerns in the OR’. Participants responded to the items on a 5-point Likert scale from 1 = ‘Strongly disagree’ to 5 = ‘Strongly agree’.

A subset of the study participants also responded to hypothetical scenarios, listed below, reporting their likelihood of speaking up on a 5-point Likert scale, where 1 meant ‘extremely unlikely’ and 5 ‘extremely likely’. Responses to each of the following hypothetical scenarios served as the dependent variable used in the ordinal regression analyses.

#### Scenario 1

During a surgery you notice inappropriate patient management that could result in an adverse outcome. The rest of the team seems unaware. Please indicate how likely you are to speak up if you are ‘*pretty sure’* that this will result in an ‘*adverse outcome’*.

#### Scenario 2

During surgery you notice inappropriate patient management that could result in an adverse outcome. The rest of the team seems unaware. Please indicate how likely you are to speak up if you are ‘not sure’ whether or not this will result in an ‘adverse outcome’.

#### Scenario 3

You are in the maintenance phase of the anaesthetic for a laparoscopic cholecystectomy in an otherwise healthy, morbidly obese, 40-year-old patient. You are in steep Trendelenburg position and the patient’s arm has fallen off the arm board three times now, which you have easily managed to notice and re-secure to the arm board each time. If faced with a similar situation, how ‘likely are you to say something to the surgeon’?

### Statistical analysis

First, exploratory factor analysis (EFA) was conducted to identify the underlying factor structure for the intrapersonal factors scale (IPFS). Based on the EFA results, composite factor subscale scores were calculated. Next, to evaluate the validity of the newly developed instrument, we tested two separate hypotheses using the IPFS subscale scores. We tested the hypothesis that the IPFS factor scores would increase with the training level. A set of three one-way ANOVAs was conducted with each IPFS factor composite score as the dependent variable and training level (PGY2, PGY3, and PGY4, or faculty) as the independent variable. Additionally, using multiple ordinal regressions with a Logit link, we examined whether the response variable, which reflects the likelihood of speaking up in three different hypothetical conditions, is explained by demographic variables (training level and gender, which were entered in the regression model as factors), or intrapersonal variables (IPFS factor scores, which were entered in the regression model as covariates).

All statistical analyses were carried out using Statistical Package for the Social Sciences (IBM SPSS Statistics for Mac, Version 25.0. Armonk, NY: IBM Corp.) with a significance level set at *p* < 0.05.

## Results

Data were collected from a total of 81 participants. Of those, 60 (26 female, 34 male) were anaesthesiology trainees and 21 (10 female, 11 male) were faculty anaesthesiologists. The distribution of trainees across training levels was fairly balanced: 30% were PGY2 (*n* = 18), 32% were PGY3 (*n* = 19), and 38% were PGY4 (*n* = 23).

A subset of these participants (62) also completed all questions related to the three hypothetical linical scenarios. Of those, 51 (23 female, 28 male) were trainees and 11 (6 female, 5 male) were faculty anaesthesiologists.

### Factor analysis

As a first step we examined the factorability of the items [28]. All but one item had a correlation above 0.3 with at least one other item. Anti-image matrices showed good values of measures of sampling adequacy for all items, with correlations all above 0.7. Sampling adequacy for exploratory factor analysis was confirmed by a Kaiser-Meyer-Olkin (KMO) value of 0.8, which was above the recommended value of 0.6. Bartlett’s test of sphericity was significant (χ^2^ (190) = 902.62, *p* < 0.001). These results indicated that the 20 items in our questionnaire were suitable for exploratory factor analysis.

The participants’ responses to all 20 items were subjected to factor analysis using principal axis factoring with orthogonal varimax rotation. Initial extraction suggested a 5-factor solution explaining 69% of the variance. It was observed that the first three factors explained 57% of the variance and were theoretically meaningful. Since the remaining factors had eigenvalues slightly above 1 and an insufficient number of primary loadings, a 3-factor solution was accepted. This led to the removal of seven items. We subsequently eliminated three more items that had primary factor loadings <0.4 or cross-loadings of >0.4.

For the remaining 10 items, we repeated the principal axis factoring with orthogonal varimax rotation to investigate whether they were all well represented by one of the three factors. The resulting 3‑factor solution explained 73% of the variance, and all items were well represented. Tab. 1 summarizes the factor loading matrix for this solution.

**Table 1:** Factor loadings and communalities based on a principal axis factoring with varimax rotation for 10 items from the Intrapersonal Factors Scale for speaking-up in the OR (*n* = 81)

Item	Self-efficacy	Social outcome expectations	Assertive attitude	Communality	
When I notice an error during a surgery, I can easily determine whether or not speaking-up is warranted	0.81			0.69	
I feel confident that I can communicate patient management concerns clearly during a surgery	0.76			0.65	
I feel confident that I can recognize when something is amiss during a surgery	0.74			0.58	
I feel confident that I can express concerns efficiently without wasting anyone’s time during a surgery	0.69			0.59	
Communicating concerns in the OR will increase my colleagues’ respect of my patient care skills		0.89		0.84	
Speaking up in the OR will increase my status among the team members		0.84		0.66	
My team will view me as competent if I effectively express patient safety concerns in the OR		0.80		0.74	
I tend to hesitate expressing opinions that may be contrary to what others think in the OR^a^			0.78	0.67	
I think it is better to defer to the senior staff rather than voicing concerns in the OR^a^			0.58	0.37	
Likelihood of me speaking up depends on my familiarity with the surgical case^a^			0.47	0.35	
Eigenvalue	4.01	1.90	1.35		
% of Total variance	40.14	18.92	13.61		
Total variance			72.67%		

^a^ Denotes a reversed scored item

Note. Factor loadings <0.35 are suppressed

Review of the items that were included under each factor within this new 3‑factor solution led to the labelling of factor 1 as ‘self-efficacy’ (Cronbach’s α = 0.86), factor 2 ‘social outcome expectations’ (Cronbach’s α = 0.89), and factor 3 ‘assertive attitude’ (Cronbach’s α = 0.67). We created composite scores for each factor using the mean of all items included within the respective factors. After reverse scoring items included under the assertive attitude factor, high scores for each factor indicate greater self-efficacy, social outcome expectation, and assertive attitude. Tab. 2 depicts descriptive statistics and reliability for the subscales.

**Table 2:** Descriptive statistics for the Intrapersonal Factors Scale for speaking-up in the OR (*n* = 81)

	# of items	*M* (*SD*)	Skewness	Kurtosis	Cronbach’s α
Self-efficacy	4	4.14 (0.61)	−0.50	−0.09	0.86
Social outcome expectations	3	3.86 (0.77)	−0.31	−0.16	0.89
Assertive attitude	3	2.88 (0.91)	−0.05	−0.58	0.67

Overall, these analyses suggested that there are three distinct factors underlying participants’ responses to the 10 items in the IPFS for speaking up in the OR. These factors were internally consistent. An approximately normal distribution was observed for the composite subscale score data in the current study; thus, the data were well suited for parametric statistical analyses.

### Hypothesis testing

#### 1. Comparison of IPFS composite scores by training level

**Fig. 1:**
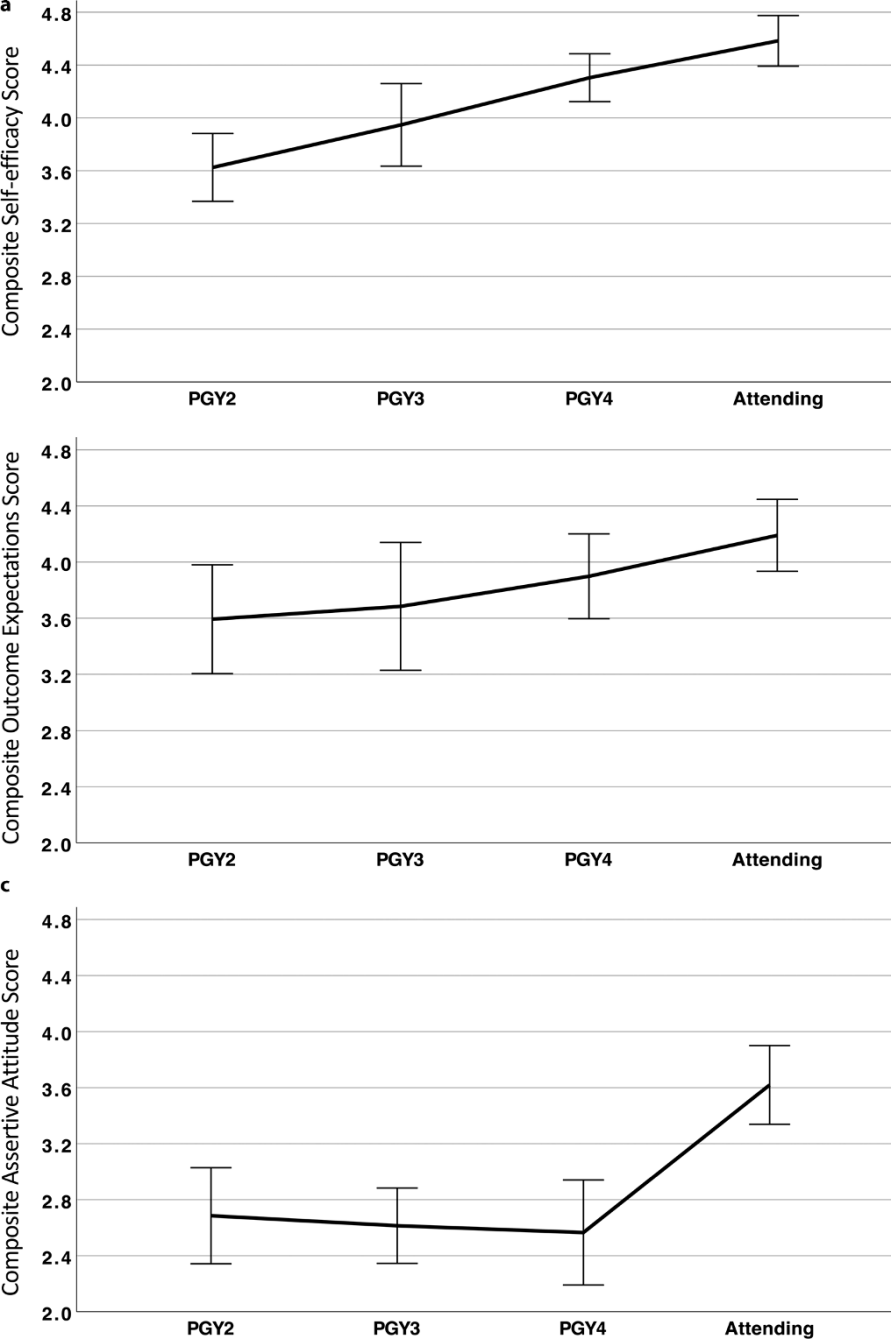
Line graph showing composite scores for intrapersonal factors by training level. Self-efficacy (**a**), social outcome expectations (**b**), assertive attitude (**c**). Error bars indicate 95% confidence intervals

Training level (PGY2, *n* = 18; PGY3, *n* = 19; PGY4, *n* = 23; faculty anaesthesiologist, *n* = 23) was significantly associated with self-efficacy (F(3, 77) = 13.45, *p* < 0.001, η^*2*^ = 0.34) and assertive attitude scores (F(3, 77) = 10.69, *p* < 0.001, η^*2*^ = 0.29), but not with social outcome expectation score (F(3, 77) = 2.48, *p* = 0.07, η^*2*^ = 0.09). Fig. 1 shows the IPFS composite subscale scores (self-efficacy Fig. 1a, social outcome expectations Fig. 1b, assertive attitude Fig. 1c).

Post-hoc analyses using the Bonferroni procedure indicated that faculty anaesthesiologists (*M* = 4.58, *SD* = 0.42) had significantly higher self-efficacy than PGY2 (*M* = 3.63, *SD* = 0.52) and PGY3 (*M* = 3.95, *SD* = 0.65) residents did; PGY4 (*M* = 4.30, *SD* = 0.42) residents had higher self-efficacy than PGY2 residents did. As for assertive attitude, faculty anaesthesiologists had significantly higher scores (*M* = 3.62, *SD* = 0.62) than PGY4 (*M* = 2.57, *SD* = 0.87), PGY3 (*M* = 2.61, *SD* = 0.56), and PGY2 (*M* = 2.67, *SD* = 0.69) residents did. No significant differences were observed between different levels of trainees in terms of assertive attitude composite scores.

#### 2. Associations between speaking up behaviour and intrapersonal factors

All multiple ordinal regression models showed a good fit (*p* < 0.001), the assumptions for parallel lines (*p* > 0.05) and goodness-of-fit were met, and both Pearson and Deviance were non-significant (*p* > 0.05). Statistically significant effects for each of the clinical scenarios are presented below.

##### Scenario 1

When the participants were ‘*pretty sure*’ that there would be an adverse patient outcome*:*

The proportional odds model showed a significant positive effect for self-efficacy (*β* = 3.706, *SE* = 0.984) and assertive attitude (*β* = 1.412, *SE* = 0.566). Nagelkerke *R*^*2*^ = 0.63.

An increase in *self-efficacy* composite score by one unit (a value between 1 and 5) was associated with an increase in the odds of speaking up, with an odds ratio of 40.772 (95% CI 5.918 to 280.058), Wald χ2(1) = 14.188, *p* < 0.001. In other words, in this scenario, the odds of speaking up for those who report one unit higher self-efficacy is about 40 times higher compared with those reporting lower self-efficacy.

An increase in *assertive attitude* composite score by one unit (a value between 1 and 5) was associated with an increase in the odds of speaking up, with an odds ratio of 4.104 (95% CI 1.352 to 12.453), Wald χ2(1) = 6.220, *p* = 0.01. This means that the odds of speaking up for those who report a single unit higher assertive attitude is about 4 times higher compared with those reporting lower assertive attitude.

##### Scenario 2

When the participants were ‘*not sure’* whether there would be an adverse patient outcome:

The proportional odds model showed a significant negative effect for training level between PGY4 and attending faculty anaesthesiologist, which was a reference category, (*β* = −2.278, *SE* = 1.024). There was a positive effect for self-efficacy (*β* = 2.440, *SE* = 0.635) and assertive attitude (*β* = 1.472, *p* = 0.001, *SE* = 0.436). Nagelkerke *R*^*2*^ = 0.62.

Attending anaesthesiologists were 8.8 times more likely to speak up than PGY4 residents, a statistically significant effect, Wald χ2(1) = 5.525, *p* = 0.033. No significant difference was observed between other training levels.

An increase in *self-efficacy* score by unit was associated with an increase in the odds of speaking up, with an odds ratio of 11.472 (95% CI 3.308 to 39.785), Wald χ2(1) = 14.786, *p* < 0.001. Similarly, an increase in *assertive attitude* score by one unit was associated with an increase in the odds of speaking up, with an odds ratio of 4.357 (95% CI 1.855 to 10.236), Wald χ2(1) = 11.404, *p* = 0.001.

##### Scenario 3

You are in the maintenance phase of the anaesthetic for a laparoscopic cholecystectomy in an otherwise healthy, morbidly obese, 40-year-old patient. You are in steep Trendelenburg position and the patient’s arm has fallen off the arm board three times now, which you have easily managed to notice and re-secure to the arm board each time. If faced with a similar situation, how ‘*likely are you to say something to the surgeon*’?

The proportional odds model showed a significant negative effect for gender between female and male, reference category, (*β* = −1.053, *SE* = 0.525). There was a positive effect for social outcome expectations (*β* = 1.771, *SE* = 0.425). Nagelkerke *R*^*2*^ = 0.50.

Males were 2.9 times more likely to speak up in this scenario than females, a statistically significant effect, Wald χ2(1) = 4.028, *p* = 0.045.

One unit increase in *social outcome expectations* score was associated with an increase in the odds of speaking up, with an odds ratio of 5.876 (95% CI 2.552 to 13.517), Wald χ2(1) = 17.345, *p* < 0.001. That is, those who report a single unit higher social outcome expectancy have 4 times higher odds of speaking up in this scenario compared with those reporting lower social outcome expectations.

## Discussion

The primary objective of this study was to create a psychometrically sound tool to measure intrapersonal factors that influence speaking up behaviour in the OR. Using EFA, we developed a 10-item questionnaire, entitled the Intrapersonal Factors Scale (IPFS) for speaking up in the OR. Our analysis suggests that the variance in the data was largely attributable to three major dimensions: self-efficacy, social outcome expectations, and assertive attitude.

To provide further validity evidence for the scale-criterion relationships [29], we also tested two hypotheses using the factors that emerged in EFA. The IPFS subscale scores indicated that respondents’ self-efficacy and assertive attitude as related to speaking up increased with training level. The association between training level and social outcome expectations scores demonstrated a nonsignificant trend. Additionally, all subscales were associated, in some capacity, with the likelihood of speaking up across three different hypothetical OR scenarios. While our findings related to intrapersonal factors, training level, gender, and speaking up behaviour are worth discussing in light of previous research, they should be interpreted with caution since they were obtained in the context of scale development.

For instance, a study by Lyndon and colleagues (2012) showed that the likelihood of speaking up for various labour and delivery scenarios correlated with bravery and assertiveness, which were associated with age and years of clinical experience [17]. Their findings also indicated that perception of harm to patient and specialty experience both predicted the likelihood of speaking up when controlling for bravery and assertiveness. Our findings also suggest that assertive attitude scores along with self-efficacy scores were associated with level of training.

Using live simulations requiring speaking up within acute care team settings, Weiss et al. (2014) showed that agency (assertiveness and persistence) was a positive predictor of speaking up, whereas communion (concerns for negative impact of speaking up on social relationships) was a negative predictor of speaking up behaviour [14]. Similarly, our results suggested that assertive attitude and positive social outcome expectations predicted speaking up behaviour in hypothetical scenarios.

These are not surprising results. As individuals progress through training, expertise is acquired and hierarchy in the OR naturally narrows. These changes through time presumably lead to practitioners becoming more confident and developing assertive attitudes toward speaking up. Although the individuals’ hierarchical status within an OR team is difficult to modify, educational interventions targeting intrapersonal factors may help expedite the development of speaking up attitudes. These interventions could utilize methods like the ‘two-challenge rule’ [7], ‘advocacy-inquiry communication’ [9], and ‘Stop, Notify, Assess, Plan, Prioritize, and Invite ideas (SNAPPI)’ [30] as well as others described by Weller and colleagues (2014) [31]. The new scale could provide a helpful measure to benchmark and evaluate such educational efforts.

Moreover, our results also showed that male gender, along with higher positive outcome expectations, was associated with increased odds of speaking up to a surgeon regarding a patient’s arm repeatedly falling off the arm board during a surgery. Studies using Big Five personality traits model [32] indicate that women value social affiliations more [33] and tend to be more polite and agreeable [34]. Our finding, though in line with personality traits research, needs further confirmation with a larger sample in a real-life healthcare setting, as professional role responsibility and patient safety concerns might attenuate or amplify this gender difference.

As social cognitive theory posits, individuals learn in social contexts. If junior members of the healthcare team are provided opportunities to practice in a safe environment while addressing underlying individual factors for speaking up behaviour, they may be more likely to overcome various other environmental challenges, and speak up in order to help improve patient outcomes.

As culture plays a role in the predisposition to speak up, this IPFS scale needs to be evaluated at other sites and among other disciplines (e.g. surgery, nursing). Moreover, in this study we used hypothetical clinical scenarios; future studies would ideally test findings in real-life speaking up behaviour or at least utilize high-fidelity simulation. Further evidence should also be considered in terms of comparing IPFS scores with other similar scales. While we were not able to identify other published scales that contained the three factors included in IPFS, a combination of the scales used in the Lyndon et al. (2012) study such as Safety Attitudes Questionnaire, the Bravery and Assertiveness subscales from the International Personality Item Pool [17] could be used to establish further validity evidence for IPFS.
